# Romanian National premiere with the new Thulium SuperPulsed Laser in the endourological treatment of urolithiasis

**DOI:** 10.25122/jml-2021-0103

**Published:** 2021

**Authors:** Bogdan Geavlete, Razvan Multescu, Valentin Iordache, Petrisor Geavlete

**Affiliations:** 1. Sanador Hospital, Bucharest, Romania; 2. Department of Urology, Sf. Ioan Emergency Clinical Hospital, Bucharest, Romania

**Keywords:** laser energy, Soltive TFL, stone fragmenting, Thulium laser, urinary stones

## Abstract

In this paper, we aimed to verify the efficiency and functionality of the new Soltive Thulium Fiber Laser (TFL) in the treatment of urinary stones. The Soltive Laser System was used in 17 urolithiasis cases: 10 renal, 5 ureteral, and 2 bladder stone patients. The average stone size was 13.1 mm (ranging between 11–29 mm) for the kidney, 8 mm (ranging between 6–12 mm) for the ureter, and 31 mm (ranging between 27–34 mm) for the bladder. Only single calculi patients were included in the study. We used 100 and 150 μm core-diameters fibers (CDF). Three settings were applied: 0.15 J/100 Hz for “fine dusting”, 0.5 J/30 Hz for “dusting” and 1 J/15 Hz for the fragmentation mode. The mean operative time was 34 minutes for renal, 21 minutes for ureteral, and 39 minutes for bladder stones. The visibility was optimal in all cases. The stone-free rate at 1 month after treatment was 95% for renal calculi and 100% for ureteral and bladder stones. Very fine dust made of stone fragments smaller than 1 mm in size that passed spontaneously through the access sheath was observed, especially subsequent to the “fine dusting” mode. The complication rate was very low: 1 patient was classified as Clavien grade 1 and 1 patient as Clavien grade 2, and this was the case for renal stones only. No urinary tract infections were observed. The new Soltive TFL appears to be a remarkably promising tool in the therapeutic approach of urolithiasis. Lithotripsy was achieved up to the level of extremely small stone fragments with improved efficiency while also optimizing the operative time.

## INTRODUCTION

At present, the Holmium laser is considered the most effective lithotripsy system and the optimal standard for the retrograde ureteroscopic approach [1]. The main reasons for that are represented by the good safety profile, effectiveness in all stone compositions well as the fact that it can also be used for tissue incision/ablation. Various pathways to improve it (such as new generators with the Moses effect) were developed during the last decade.

However, a new type of energy source for lithotripsy seems to be now challenging this position. Thulium fiber laser (TFL) operates at 1940 nm, operating a silica fiber that includes Thulium ions. Therefore, it must be clarified that Holmium and Thulium are two distinct chemical elements with 67 and 69 protons in their nucleus, respectively, and have been classified as rare-earth elements in the periodic table. Similar to other rare-earth ions, trivalent Holmium and Thulium ions have a unique set of emission wavelengths, particularly in the near-infrared range. These ions are excited by multiple diode lasers, while laser energy can be applied either in a continuous or a pulsed manner [2]. TFL must not be confused with the Thulium:YAG laser, which is a solid-state laser, similar to the Holmium:YAG laser, and operates at a 2010 nm wavelength.

An article entitled “Real-world study launches of super-pulse thulium fiber laser system for urinary stones” by Jason M. Broderick was published in Urology Times on January 7, 2021. Recently, he announced that the first patient had been enrolled in a real-world study of the SoltiveSuperPulsed Laser System (Soltive Laser System), which uses Thulium fiber laser technology to disintegrate kidney and ureteral stones (it is important to mention that the first patient in the real-world study was treated by Wilson Molina, MD, Professor of Urology at the University of Kansas School of Medicine at the end of 2019).

The aim of the present analysis was to review the initial experience of our clinical department with Soltive TFL (one of the first in the world) used for urolithiasis treatment, both in the upper and lower urinary tract.

## MATERIAL AND METHODS

**Figure 1:**
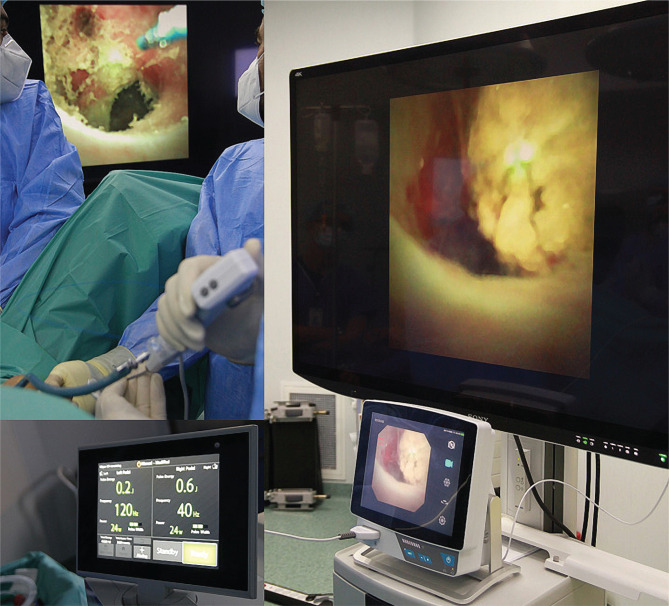
Intraoperative aspects of Soltive lithotripsy of a large renal stone (2.5/1.7 cm) disintegrated with this new Thulium fiber laser system. We used a single-use digital flexible ureteroscope (Pusen PU 3022A) and the Sony 55-inch monitor (double-checked image with the original Pusen monitor).

As part of these initial series, Soltive TFL was used in 17 cases of stone disease: 10 renal, 5 ureteral and 2 bladder stone patients. The average stone size was 13.1 mm (ranging between 11–29 mm) for the kidney, 8 mm (ranging between 6–12 mm) for the ureter, and 31 mm (ranging between 27–34 mm) for the bladder. Only single calculi patients were included in the study (Figure 1).

All patients had preoperative computed tomography (CT) scans, the average stone density being 926 Hounsfield units (HU) for renal stones (ranging between 430–1752), 911 HU for ureteral stones (ranging between 410–1700), and 1240 HU for bladder stones (ranging between 970–1510). In all cases, only solitary stones were treated.

**Figure 2:**
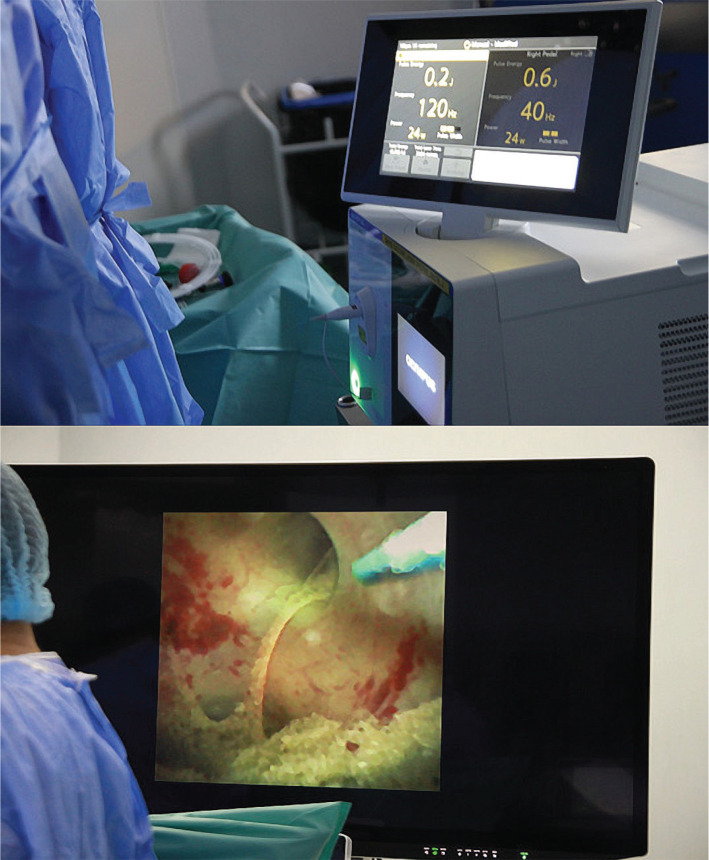
Intraoperative settings of the Soltive system during lithotripsy – dusting and fine dusting disintegration. Impressive images on the Sony monitor with complete stone dusting.

For lithotripsy with Soltive TFL, 100 and 150 μm core-diameters fibers (CDF) were used. The settings applied were 0.15 J/100 Hz for “fine dusting”, 0.5 J/30 Hz for “dusting” and 1 J/15 Hz for “fragmentation”. Previously, in the case of hard stones, “fine dusting” lower pulse energy (0.025–0.15 J) and higher pulse rate (40–2000 Hz) were used instead of the standard “dusting” mode of the Ho:YAG laser, using the minimum energy of 0.2 J (Figure 2).

For renal stones, we used disposable ureteroscopes (PU3022A, Pusen, Zhuhai, China); for ureteral calculi, we used the Storz 10Fr semirigid ureteroscopes and the 21Fr Storz rigid cystoscope for bladder stones. We retrospectively analyzed the operative time and intraoperative features (including visibility and retropulsion) as well as complications associated with all procedures.

## RESULTS

The mean operative time to complete stone disintegration (up to the state of dust) was 34 minutes for renal stones, 21 minutes for ureteral stones, and 39 minutes for bladder stones.

We also analyzed retropulsion during laser lithotripsy. Significant retropulsion was defined as the situation in which supplementary maneuvers are necessary auxiliary to the movements of stone fragments during laser application (such as relocation of stone fragments using baskets). It was found that retropulsion was insignificant in all cases with energy levels equal to or less than 0.5 J.

**Figure 3:**
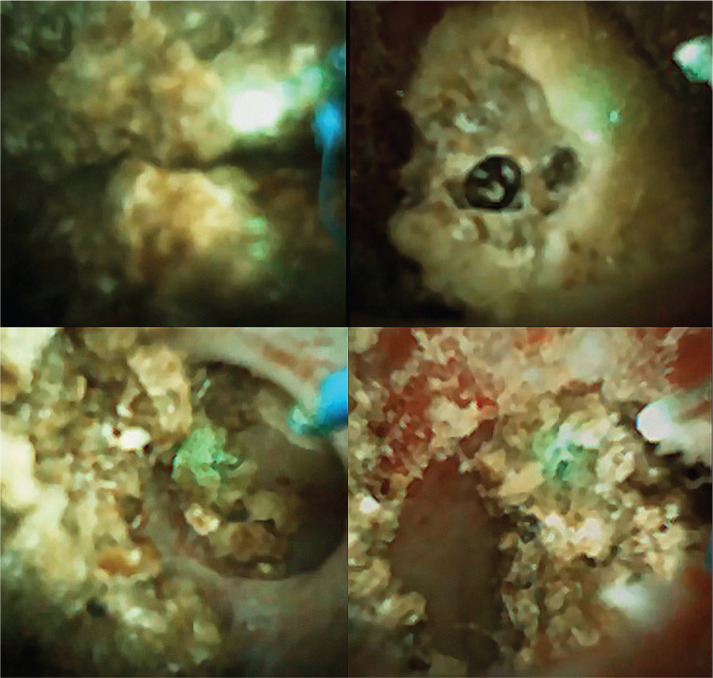
First images of different lithotripsy moments using the Soltive system (fragmentation and dusting). 150 μm core-diameters fibers (CDF).

The operator assessed visibility using scores of 1 (poor visibility, which does not allow continuation of the procedure), 2 (poor visibility, which requires further measures to be able to continue the procedure), 3 (average visibility, prolonging the operative time), 4 (slightly impaired visibility without any impact on the operative time) or 5 (excellent visibility). Scores of 4 or 5 were attributed in all cases (Figure 3).

**Figure 4:**
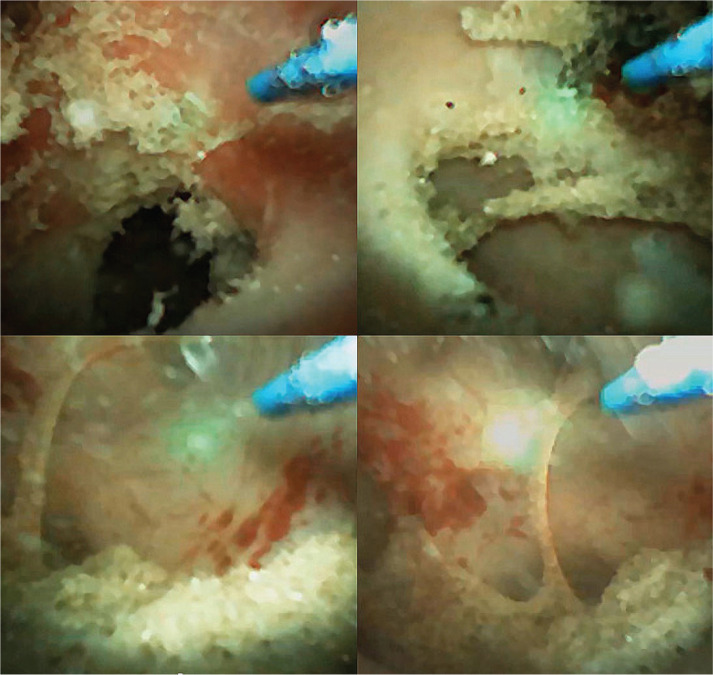
Final aspects of a real fine dusting of the stone (Soltive TFL – 0.15 J/100 Hz).

Very fine dust, stone fragments of less than 1 mm in size, susceptible to pass spontaneously through the access sheath alongside the scope, were observed, especially in the “fine dusting” mode (0.15 J/100 Hz). Also, the size of stone fragments allowed aspiration through the working channel of the ureteroscope (Figure 4).

The complication rate was very low: 1 case was classified as Clavien grade 1, and 1 patient was classified as Clavien grade 2 for renal stones only. We did not find any cases of Clavien grade 3, 4 or 5 complications. Moreover, no urinary tract infections were observed.

The stone-free rate at 1 month after treatment was evaluated by kidney, ureter, and bladder (KUB) x-rays in 11 cases (8 with kidney and 3 with ureteral stones), by CT scan in 4 cases (2 with renal and 2 with ureteral calculi) and by ultrasound for both bladder stone cases: 95% for renal stones and 100% for ureteral and bladder stones.

## DISCUSSION

The initial data for TFL are extremely promising. TFL wavelength is 1940 nm, this being the infrared absorption peak of water at 22°C. As water heats, this peak is shifting towards 1920 nm at 70 degrees [3]. Optical penetration in the water of TFL, being about four times shorter than that of Holmium laser, may offer, at least theoretically, a four-times lower stone ablation thresholds as well as an improved tissue ablation efficacy [4].

TFL offers frequencies much higher than Holmium (up to 2200 Hz) and very low pulse energies (down to 0.005 J), the combination of these settings providing the conditions for a “fine dusting” mode to be achieved, which is not available for the Holmium laser [5]. This kind of lithotripsy generates tiny stone fragments that can be evacuated more easily, even when using suction devices. It creates more opportunities to manage residual lithiasis material after lithotripsy, especially in large stones.

TFL employs small-caliber fibers (150 or even 50 microns), which have a reduced influence of deflection, retropulsion, and irrigation by comparison to the larger Holmium laser fibers (270 microns). This aspect translates into better maneuverability and vision and, therefore, superior performances and safety. Also, in the long run, this feature may create the circumstances for further miniaturization of the endoscopes.

In one of the first real-life setting studies, TFL was confirmed as an effective lithotripsy device to be used during the flexible retrograde approach of lower pole stones. It allowed access to all inferior calyceal stones, even when acute infundibulo-pyelic angles were present [6].

During the present study, this therapeutic approach was shown to be a safe and effective lithotripsy method for bladder, ureteral and renal calculi.

One of the main advantages of Holmium laser is that it can be successfully used for lithotripsy regardless of the stone composition. Similarly, in a study evaluating TFL for ureteral calculi treatment using the retrograde ureteroscopic approach, this tool proved to be a safe and effective lithotripsy method regardless of stone density, similarly to Holmium laser [7].

Kronenberg and Traxer described a reduced retropulsion or no retropulsion for the new TFL, adding this to the improved performances [4]. In another real-life setting study analyzing TFL during percutaneous surgery, retropulsion was described as interfering with surgery in 1.7% of the cases, and insignificant retropulsion was noted in 10.8% of cases. In the same analysis, poor visualization was reported in 2.5% of the cases and minor difficulties regarding visibility in 3.3% of the cases [8]. Similarly, no significant retropulsion was recorded in the present study when the energy level was maintained under 0.5 J.

Another parameter to be analyzed is the temperature increase when the laser is operated. An in vitro study by Taratkin *et al*. compared TFL with Holmium laser at 0.2 J and 40 Hz, fired for 60 seconds with and without irrigation flow. The authors concluded that there are no significant differences in terms of volume-averaged temperature increase. Irrigation reduced the temperatures for both lasers similarly: TFL – 1.9°C; Ho:YAG laser – 2.8°C (with 35 ml/second at 60 seconds) [9].

In a similar manner, Peng *et al*. evaluated the thermal effect of TFL and demonstrated it to be safe to use as long as there is moderate irrigation while also considering that it may be necessary to decrease the power if irrigation is ceased [10].

It may be useful to underline the recent findings (2021) of Ben Chew, MD, MSc, Associate Professor in the Department of Urologic Sciences at the University of British Columbia in Vancouver and co-principal investigator of the world first trial (stated in a press release): “the Soltive Laser System has a completely different way of delivering the laser energy to the stone, being now able to fragment stones very quickly and into much smaller pieces, like the fine sand you would find at a white sandy beach”.

In fact, our initial experience, despite the reduced number of cases, sustains the following findings: “with Soltive, urologists may be able to treat bigger stones endoscopically, so the indication of the percutaneous approaches could be dramatically reduced”. Also, it was noticed in the present analysis that this procedure remains efficient and safe, even for large stones.

Moreover, the already proved advantages of this new IPG Protopype Superpulse Tm Fiber System (Soltive laser) versus Lumenis Pulse 120H (Ho long), one of the latest and most advanced Holmium lasers, were systematized by Traxer [12]:

1. Pulse Energy (PE), measured in J: 10 times less;
2. Max Peak Power, W: 4 times less;
3. Max Repetition Rate, Hz: 25 times more;
4. Fibers: 4 times smaller (16/surface);
5. Laser Beam Profiles at the Surgical Fiber Connector: 16 times smaller;
6. Ho: YAG Laser with Moses vs. Super Pulse Thulium Fiber Laser: 2 times more dusting.

Nowadays, another main advantage of TFL lasers appears to be represented by the greatest mean diameter of fragments produced, which is limited to 111.9±189.8 μm. Therefore, the definition of dust could be changed: particles that can be aspirated through the working channel of a ureteroscope or stone fragments <1 mm in size that pass spontaneously, floating small particles created merely by using irrigation during the ureteroscopy, analogous to a snow globe [11].

## CONCLUSION

According to the present initial experience, this new TFL (Soltive) laser constitutes a remarkably promising lithotripsy tool, potentially challenging the Holmium laser’s position as the most effective lithotripsy method benefitting from the “standard” status.

With a completely different way of delivering the laser energy to the stone, it seems possible to fragment stones more quickly and into much smaller pieces; so, in other words, more dust and much faster. Among other benefits, the Soltive Laser System also demonstrated an extremely low level of retropulsion.

## Data Availability

The datasets generated and/or analyzed during the current study are available by request.
